# In which US counties are anaerobic dairy digesters being implemented?

**DOI:** 10.3168/jdsc.2023-0385

**Published:** 2023-08-12

**Authors:** J.K. O'Hara, I.M. Xiarchos

**Affiliations:** Office of the Chief Economist, US Department of Agriculture, Washington, DC 20250

## Abstract

There has been limited research on the recent increase in US anaerobic dairy digesters. We use panel data to estimate a double hurdle model that predicts both the probability that a county has a dairy digester and the number of digesters in counties that have them. Dairy farms and retail electricity prices affect dairy digesters, although the parameter estimates are inelastic. Biogas-producing landfills, which reflect policies and infrastructure that enable renewable energy, also affect digesters. Our findings on the conditions that are conducive to dairy digesters will help policymakers consider options for reducing manure methane emissions.

## Highlights

• Digesters increase modestly from increases in either small or large dairy farms.
• The likelihood of a digester increases from more electricity-generating landfills.
• Digesters increase from increases in renewable natural gas landfills.
• Proximity to natural gas pipelines does not influence digester installation.

## Graphical Abstract

**Figure:**
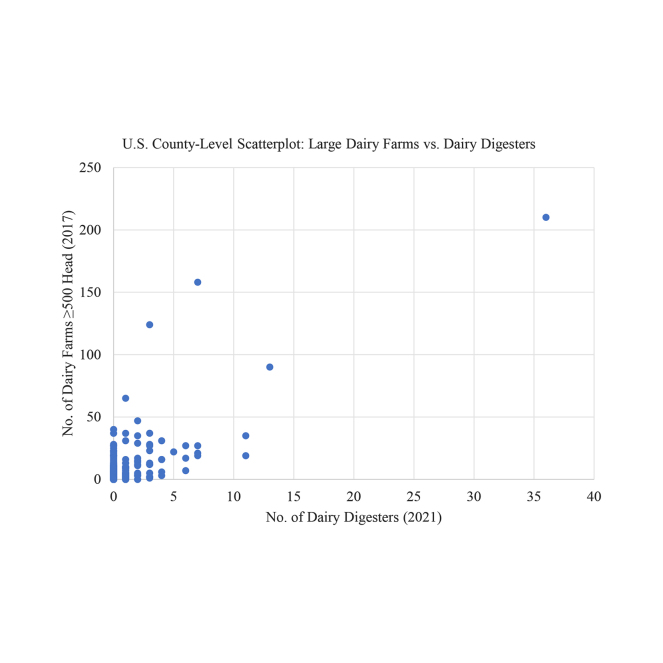
**Summary:** County-level studies on the characteristics conducive for dairy digesters will benefit policy efforts to reduce manure methane emissions. Regional studies are needed in the United States because newer digesters are collecting manure from multiple farms and are increasingly injecting biogas into pipelines for use as natural gas. We evaluate how the number of dairy farms and proximity to biogas infrastructure influenced the likelihood of a dairy digester in US counties over time, as well as the number of dairy digesters. We found that both small and large dairy farms influence digesters, although the effects are modest. Similarly, higher retail electricity prices have a modest effect on digester adoption. While we found an association between biogas-producing landfills and digesters, we did not find a relationship between natural gas pipeline proximity and digesters.

Anaerobic digesters can capture manure CH_4_ emissions that would otherwise be released into the atmosphere. In addition to the environmental benefits that digesters provide, US policies promote them (a) because using biogas for renewable energy production provides farms with byproduct revenue and (b) domestic biogas production enhances energy security. In the United States, 84% of the 341 livestock digesters collected manure from dairy farms in 2021 (EPA, 2022b).

Understanding the characteristics of counties that are conducive for dairy digesters would help US policymakers develop plans that reduce manure CH_4_ emissions.

However, there have been few county-level studies of US dairy digesters to date. Instead, much of the anaerobic digester research has focused on how digester policies affect the profitability of a digester on a representative farm that produces electricity with biogas. These studies include Lazarus and Rudstrom (2007), Bishop and Shumway (2009), Key and Sneeringer (2011, 2012), Wang et al. (2011), and Cowley and Brorsen (2018a,b). While studies have compared the effectiveness of incentives for digesters across broad US geographic regions (e.g., Key and Sneeringer, 2011), county-level analyses historically have been infeasible given the low deployment of digesters. County-level studies were also less meaningful historically given that (a) older digesters are smaller and unlikely to collect manure from multiple farms and (b) older digesters tended to use biogas to produce electricity, which implies that proximity to natural gas pipelines was unimportant.

One objective is this research is to understand, at the county level in the United States, how the number of dairy farms influenced the probability of a dairy digester and, among counties with digesters, the number of dairy digesters. Understanding how the number of dairy farms affects digesters has become important because digesters are becoming increasingly larger (Figure 1). In particular, clusters of farms can influence digester installation since larger digesters collect manure from multiple farms. So, while digester installation is influenced by the concentration of livestock farms, this issue has not been explored in past studies.

**Figure 1:**
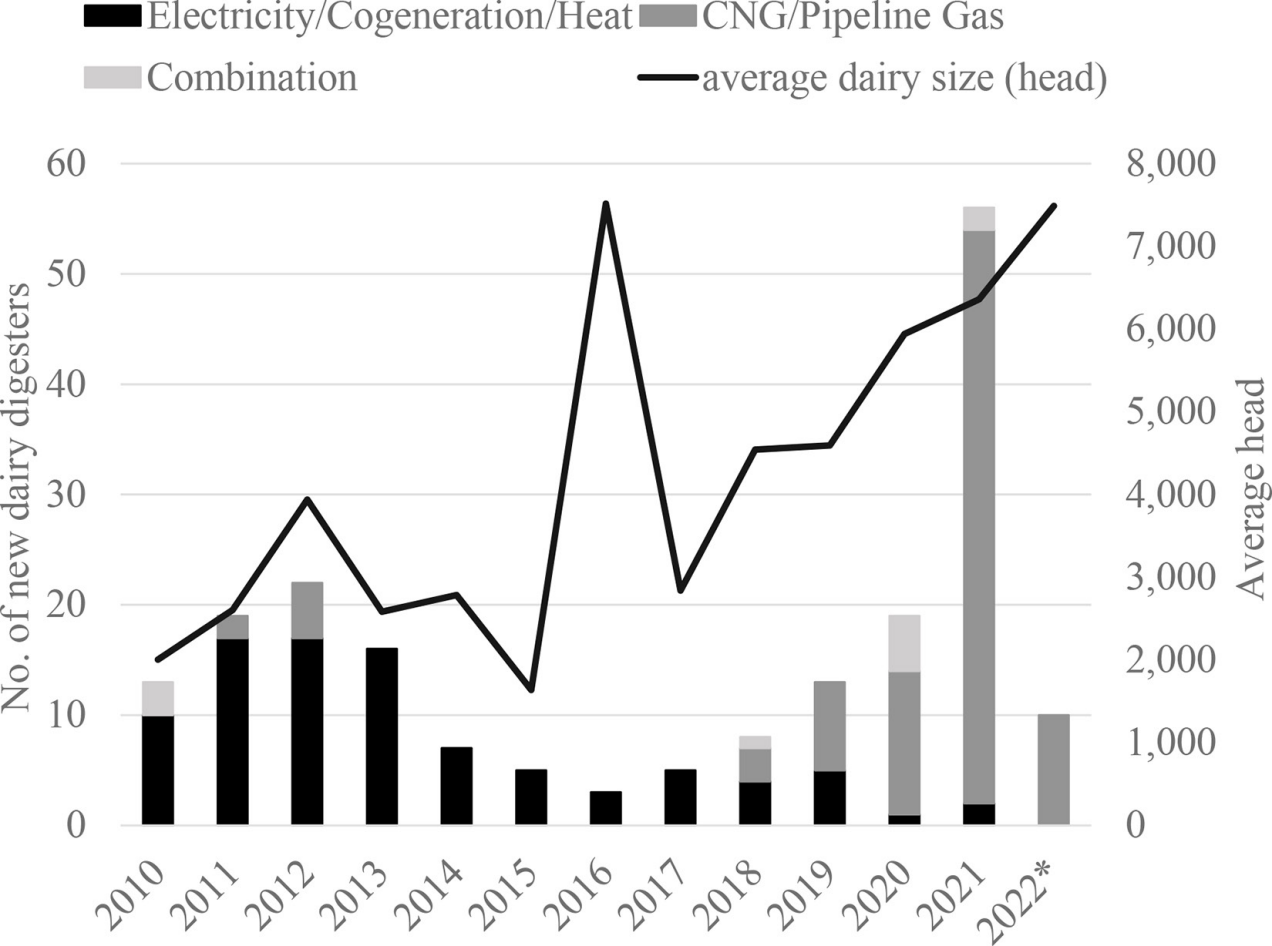
Dairy digester additions by size and biogas end-use: 2010–2022 (source: EPA, 2022a). *2022 data are not final. The data in the figure represent digesters that are currently operating on dairy farms without other livestock types.

A second goal of our study is to understand the county-level association between biogas infrastructure and dairy digesters. Understanding this relationship has become important since the recent growth in digesters has predominantly come from those that produce compressed or renewable natural gas (**CNG** or **RNG**) instead of electricity (Figure 1; Smith, 2022). Given that the feedstock or biogas can be collected from numerous farms, installation may be increasingly influenced by natural gas pipeline proximity and other biogas infrastructure.

We estimate a “double hurdle” model to investigate county-level trends in both the presence and number of dairy digesters. We use a double hurdle model, instead of a Tobit model, to test how changes in dairy farms and other exogenous variables affect the presence of dairy digesters separately than the impact of these independent variables on the number of digesters.

The first-tier equation in a double hurdle model is

[1] $$P\left(y_{2}=1|x\right)=\Phi \left(x\gamma \right),$$

where Φ is the standard normal cumulative distribution function in [1], *y*_2_ is a binary variable that equals 1 if a county has at least one dairy digester and equal to 0 otherwise, and *x* represents the independent variables. We estimate the parameters *γ* using a probit model. These parameter estimates reflect how their corresponding independent variables affect the probability that a county has at least one dairy digester. We estimate [1] among US counties with at least one dairy farm, as it is not possible for dairy digesters to exist otherwise. We estimate this regression across 3 time periods: 2011, 2016, and 2021.

The second-tier equation in a double hurdle model is

[2] $$log\left(y_{1}\right)=x\beta +\varepsilon .$$

The dependent variable in [2], *y*_1_, is the number of anaerobic digesters in a county that collect all or some manure from dairy cattle. We estimate the parameters *β* in [2] using ordinary least squares (**OLS**) among the subset of counties that have at least one dairy digester in 2011, 2016, or 2021. The parameter estimates in [2] represent the effect of these independent variables on the level of digesters in a county. Since we are using the natural log of digesters as the dependent variable, the parameter estimates *β* represent the percentage change in digesters resulting from a 1-unit increase of their corresponding variable.

We have an unbalanced panel in both [1] and [2] (i.e., some counties are represented once or twice throughout the 3 time periods). This occurs because, for instance, it is possible for dairy farms to exist in a county in one year but not in another year or a county may have an anaerobic digester in 2021 but not in 2011 or 2016. In both regressions [1] and [2], we include the same independent variables and cluster robust standard errors at the county level.

Our source for digesters per county is the AgSTAR Livestock Anaerobic Digester Database (EPA, 2022b). AgSTAR maintains the database through tracking news articles, press releases, data provided by government agencies, and outreach with industry representatives and updates it twice a year. The database contains details such as the digester start date, shutdown date, type of animal(s) from which the manure is collected, and the digester's county. Thus, due to the nature of how the database is updated, there may be digesters operating in the United States that are not listed in the database and vice versa.

We include 2 county-level independent variables that reflect the number of dairy farms with (1) 1–499 head and (2) 500 or more head. The number of farms with milk cows by herd size for 2007, 2012, and 2017 is available from the Census of Agriculture (USDA NASS, 2022). Instead of including one variable that represents the total number of dairy farms, we create 2 disaggregated variables based on size thresholds. This is because the fixed costs associated with installing digesters imply that they are only feasible on larger dairy farms (Pape et al., 2016). So, we include the 500 or more category since it is the largest size threshold for county-level data in the Census of Agriculture and has been used as a threshold size for digesters by EPA (2018). Dairies of this size may be large enough to have their own digester, whereas smaller dairies are unlikely to do so. Still, we include the 1–499 head category because many smaller farms could increase the likelihood of a digester if manure is aggregated from smaller farms.

We align the 3 time periods when the number of farms variable is reported in the Census of Agriculture (2007, 2012, and 2017) with the number of digesters 4 years in the future (2011, 2016, and 2021, respectively). We use the lag of this independent variable since the planning and construction of a digester can take several years. Thus, an increase in dairy farms would not cause a contemporaneous increase in the number of digesters.

We include the natural gas pipeline length:area ratio as an independent variable. This “pipeline density index” represents the length of natural gas pipeline in a county (measured in kilometers) divided by the county's area (measured in square kilometers). We create this variable by deriving the length of natural gas pipeline in a county from a 2020 map (EIA 2022a). Higher values of the variable imply that there is a long pipeline length relative to the county's area, and vice versa. The pipeline density index is a proxy for the ease of access that dairy farms in a county can have to natural gas pipelines. Thus, the coefficient indicates whether there are more digesters in counties with greater pipeline accessibility. Values for the pipeline density index are not available at different points in time. However, since constructing a pipeline takes a long time, digesters might have been constructed based on the anticipation of pipeline additions up to 2020.

We include 2 county-level variables that represent the number of landfill gas energy projects. These 2 variables represent the number of landfills that use biogas to produce energy for (a) electricity or heat and (b) RNG. We obtain landfill data from a database for landfill biogas projects (EPA, 2022c), which contains details such as energy type, start date, and project county.

For similar reasons as the dairy farm variables, we also include the landfill variables as 4-year lags relative to the dairy digester variables. The expected signs of these landfill variables are ambiguous a priori. On the one hand, our pipeline variable is an imperfect proxy for pipeline access as we discussed previously. So, the landfill RNG variable could be positive if it is correlated with factors that improve the capability of biogas projects in the county to sell natural gas. On the other hand, these 2 coefficients could be negative if efforts to expand biogas from dairy digesters could come at the expense of biogas from landfills (Smith, 2022).

We control for the number of food manufacturing establishments in a county (Census Bureau, 2022). For this variable, we use County Business Pattern data that corresponds to NAICS code 311. We include this variable because “tipping fees” from accepting food waste from local food processors could be a critical revenue source for farms in operating a digester (Bishop and Shumway, 2009). A greater number of food manufacturers could increase dairy digesters when the “tipping fee” practice is widespread. Like the dairy farm and landfill variables, we use a 4-year lag of our food manufacturing establishment variable given the time entailed with constructing a digester.

We include 2 variables that reflect incentives for constructing digesters for electricity sales. The first variable is the average state-level retail electricity prices, which is measured in dollars per kilowatt hour ($/kWh) in 2020 dollars (BLS, 2022; EIA, 2022b). Since these prices fluctuate over time, we use the average prices for the 3 preceding years (e.g., the average electricity price for 2018–2020 is an independent variable when the dependent variable is the number of dairy digesters in 2021). We include this variable since it has been considered in past research (Lazarus and Rudstrom, 2007; Bishop and Shumway, 2009; Wang et al., 2011; Cowley and Brorsen, 2018a). The second variable is an indicator variable that represents whether a state recognizes agricultural manure projects in its Renewable Portfolio Standard (RPS) (Weiss et al., 2020; NCSL, 2022).

We use the rural-urban continuum codes to classify whether the counties are (a) metropolitan counties or (b) nonmetropolitan counties that are adjacent to metropolitan counties (USDA ERS, 2022). We also include dummy variables for 2016 and 2021 to control for year-specific changes that influence the presence or number of dairy digesters. These can include changes in biofuel prices or changes in digester policies. While there were relatively few US digesters implemented between 2013 and 2018, there was a 38% increase in digesters between 2018 and 2021 (EPA, 2022b).

We use regional fixed effects (i.e., indicator variables that are equal to 1 if a county is within a specified region and equal to 0 otherwise) using Climate Hub regional definitions from USDA (2021). These variables capture regional-specific characteristics that could influence the feasibility of dairy digesters. While there are other similar regional definitions, the advantages of the Climate Hub definitions in our study are that (a) California, which is the largest US dairy state and where recent digester construction is concentrated, is its own region and (b) the Climate Hubs are a climate service network that translates research findings to practitioners and stakeholders.

We report the marginal effects associated with the probit regression to make the results easier to interpret. We calculate the marginal effects for a given variable when the other variables are evaluated at their mean values. The OLS parameter estimates are the same as the marginal effects, so we do not report marginal effects separately in the second-tier regressions. We also report elasticities to assist with interpreting the OLS coefficients. Elasticities represent the percentage change in one variable divided by the percentage change in another variable.

In the first-tier regression (i.e., counties with at least one dairy farm during the 3 time periods), there are 7,431 observations across the 3 time periods representing 2,801 counties. Of these counties, 4% have dairy digesters (Table 1). There are 332 observations in the second-tier regression from 135 counties.

**Table 1:** Descriptive statistics of selected variables

Item	Counties with dairy farms	Counties with dairy digesters
Observations	7,431	332
Counties	2,801	135

	Mean	SD	Minimum	Maximum	Mean	SD	Minimum	Maximum
Presence of dairy digesters (0/1)	0.04	0.21	0	1	1	0	1	1
No. of dairy digesters	0.09	0.66	0	36	1.90	2.52	1	36
Pipeline density index (km/km^2^)	0.05	0.07	0	0.77	0.04	0.04	0	0.27
No. of dairy farms (1–499 head)	25.52	68.99	0	1,979	123.89	218.00	0	1,979
No. of dairy farms (500 or more head)	1.36	7.72	0	239	14.08	30.93	0	239
No. of food manufacturing establishments	8.31	30.54	0	1,103	21.88	32.70	0	265
No. of landfills (heat or power)	0.09	0.43	0	9	0.29	0.77	0	6
No. of landfills (RNG)^1^	0.01	0.10	0	3	0.03	0.22	0	3
Retail electricity prices ($/kWh)	10.36	2.16	5.54	21.27	12.34	3.25	7.49	21.27

1 RNG = renewable natural gas.

On average, counties with dairy digesters have 14.08 dairy farms with 500 head or greater, relative to 1.36 such farms for counties with at least one dairy farm. There are 4 counties in California (Kings, Merced, Stanislaus, and Tulare) with at least 90 dairy farms with 500 or more head in each of the 3 time periods. The maximum number of digesters—36 dairy digesters in 2021—occurs in one of these counties (Tulare County). There are more food manufacturing establishments and renewable energy landfills, on average, in counties with dairy digesters relative to counties with dairy farms (Table 1).

In Table 2, increases in dairy farms of either 1–499 head or 500 or more head increase the probability that a county has a dairy digester (*P* < 0.01). The marginal effects associated with these variables are 0.01% and 0.23%, respectively (e.g., an increase in one farm with 500 or more head increases the likelihood that a county will have a dairy digester by 0.23%). An increase in a landfill that produces heat or power increases the likelihood a county will have a digester by 0.46% (*P* < 0.01). An increase in retail electricity prices of $1/kWh leads to a 0.2% increase in the likelihood that a county will have a dairy digester.

**Table 2:** First- and second-tier dairy digester regression results^1^, ^2^

Regression type	Probit	Probit–marginal effects	OLS
Pipeline density index (km/km^2^)	−1.154	−0.040	−0.247
	(0.695)	(0.038)	(1.142)
No. of dairy farms (1–499 head)	0.0030***	0.00010***	0.0009***
	(0.0006)	(0.00003)	(0.0002)
No. of dairy farms (500 or more head)	0.066***	0.0023***	0.0053***
	(0.012)	(0.0005)	(0.0019)
No. of food manufacturing establishments	−0.0010	−0.00004	−0.0025**
	(0.0013)	(0.00004)	(0.0012)
No. of landfills (heat or power)	0.133***	0.0046***	0.009
	(0.045)	(0.0016)	(0.027)
No. of landfills (RNG)^2^	0.207	0.0072	0.263***
	(0.158)	(0.0055)	(0.073)
Retail electricity prices ($/kWh)	0.068***	0.002**	0.053**
	(0.026)	(0.001)	(0.024)

1 Regional, year, metropolitan, and Renewable Portfolio Standard (RPS) indicator variables, along with constant, are included in regression but not reported. Robust standard errors clustered at county level and reported in parentheses.

2 OLS = ordinary least squares; RNG = renewable natural gas.

*** *P* < 0.01,

** *P* < 0.05.

An increase of one dairy farm of 1–499 head is associated with a 0.09% increase in the number of dairy digesters in the second-tier OLS regression (*P* < 0.01), whereas such an increase in farms with 500 or more head is associated with a 0.53% increase in digesters (*P* < 0.01). An additional food manufacturing establishment decreases dairy digesters by 0.25% (*P* < 0.05), whereas one additional RNG landfill increases the number of dairy digesters by 26.3% (*P* < 0.01). A $1/kWh increase in retail electricity prices increases digesters by 5.3% (*P* < 0.03).

An increase in dairy farms with 500 or more head increases both the likelihood that a county has a dairy digester (among all counties with dairy farms) and the number of dairy digesters in counties with digesters. We expect these coefficients to be positive due to the economies of scale associated with dairy digesters (Pape et al., 2016). However, the effects are inelastic. For instance, a 0.53% increase in digesters in the second-tier OLS regression corresponds to an elasticity of 0.07 (using the mean number of dairy farms with 500 or more head in Table 1).

Following a similar calculation, a 0.09% increase in digesters from an increase one farm with 1–499 head corresponds to an elasticity of 0.11. One reason why the coefficient on dairy farms with 1–499 head is positive is if smaller dairy farms are purchasing digested fiber for bedding from larger dairy farms with digesters (Bishop and Shumway, 2009). A second reason is if larger dairy farms are co-digesting manure from smaller proximate farms. A third reason is that that biogas is collected from an agglomeration of farms, including smaller farms. In all scenarios dairy digesters are increasing due to the concentration of dairy farms in an area, which is something that previous studies have not explored.

The landfill heat/power coefficient is positive with significance in the first-tier regression and insignificant in the second-tier regression, and vice versa with the landfill RNG coefficient. In the second-tier regression, the landfill RNG coefficient magnitude corresponds to an elasticity of 0.01. Among all counties with dairy farms, the landfill heat/power is significant if it is a proxy for counties with a more supportable environment for renewable energy projects from biogas. One reason the landfill RNG coefficient variable is insignificant in the first-tier regression could be because only 0.01 of counties have such landfills (Table 1).

The pipeline density index coefficient in statistically insignificant in both regressions. Reasons why the index is insignificant could be (a) if a county-level index is too spatially coarse to be an effective proxy for the ability of a dairy farm to access a RNG pipeline or (b) other characteristics besides pipeline proximity are important for digester installation, including characteristics associated with the RNG landfill variable.

The positive coefficients associated with retail electricity prices are consistent with our expectations and past literature (e.g., Cowley and Brorsen, 2018a). These coefficients imply that higher electricity prices either provide greater cost savings or revenue opportunities to dairy digesters that generate electricity. In the second-tier OLS regression, the electricity price coefficient corresponds to an elasticity of 0.65.

The negative coefficient on the food manufacturing establishments in the second-tier regression is inconsistent with what we anticipated. One potential explanation is if food manufacturing establishments provide a competitive advantage to landfills if food waste from these establishments was predominantly directed to landfills.

The United States is seeking to reduce greenhouse gas emission reductions from agriculture to comply with the Paris Agreement and seeking to cut CH_4_ emissions specifically as part of the Global Methane Pledge.

While past studies have focused on the profitability of installing a digester on one farm, regional-level manure management studies are needed as digesters increasingly aggregate manure or biogas from multiple farms and require pipeline accessibility for CNG/RNG production. We undertake one of the first studies to explore county-level characteristics that influence the installation of anaerobic digesters on US dairy farms. To summarize, we find that increases in smaller dairy farms, larger dairy farms, and electricity prices have a statistically significant effect on dairy digesters. Each of these effects are inelastic, with electricity prices exhibiting the greatest elasticity among the 3 variables. Food establishments have a negative effect on the number of digesters, while RNG landfills have a positive influence on the number of digesters. The pipeline density index coefficients were statistically insignificant.

One shortcoming of our panel study is the measurement error with our pipeline density index for the 2 older time periods of 2011 and 2016. This measurement error is not a major concern since we do not expect the pipeline density index to influence digester adoption at those times periods anyway. Still, a more fundamental drawback from our approach arises from the coarseness of using county-level boundaries to assess the proximity of a group of farms to natural gas pipelines. Studies that occurred with data at more granular scales could shed further insights into where dairy farms could connect to pipelines, and thus be incentivized to install a digester for CNG production.
